# Automated non-PPE detection on construction sites using YOLOv10 and transformer architectures for surveillance and body worn cameras with benchmark datasets

**DOI:** 10.1038/s41598-025-12468-8

**Published:** 2025-07-25

**Authors:** Seunghyeon Wang

**Affiliations:** Institute for Environmental Design and Engineering, University College London, London, WC1H 0NN UK

**Keywords:** Personal protective equipment monitoring, Construction management, Computer vision, Deep learning, YOLOv10, Transformer architectures, Engineering, Civil engineering

## Abstract

Ensuring proper Personal Protective Equipment (PPE) compliance is crucial for maintaining worker safety and reducing accident risks on construction sites. Previous research has explored various object detection methodologies for automated monitoring of non-PPE compliance; however, achieving higher accuracy and computational efficiency remains critical for practical real-time applications. Addressing this challenge, the current study presents an extensive evaluation of You Only Look Once version 10 (YOLOv10)-based object detection models designed specifically to detect essential PPE items such as helmets, masks, vests, gloves, and shoes. The analysis utilized an extensive dataset gathered from multiple sources, including surveillance cameras, body-worn camera footage, and publicly accessible benchmark datasets, ensuring thorough and realistic evaluation conditions. The analysis was conducted using an extensive dataset compiled from multiple sources, including surveillance cameras, body-worn camera footage, and publicly available benchmark datasets, to ensure a thorough evaluation under realistic conditions. Experimental outcomes revealed that the Swin Transformer-based YOLOv10 model delivered the best overall performance, achieving AP50 scores of 92.4% for non-helmet, 88.17% for non-mask, 87.17% for non-vest, 85.36% for non-glove, and 83.48% for non-shoes, with an overall average AP50 of 87.32%. Additionally, these findings underscored the superior performance of transformer-based architectures compared to traditional detection methods across multiple backbone configurations. The paper concludes by discussing the practical implications, potential limitations, and broader applicability of the YOLOv10-based approach, while also highlighting opportunities and directions for future advancements.

## Introduction

On construction sites, utilizing Personal Protective Equipment (PPE)—including helmets, safety vests, gloves, masks, and shoes—is critical to preventing workplace injuries. Nonetheless, many construction workers frequently fail to adhere to PPE guidelines, often citing discomfort or insufficient awareness of safety protocols^1^. Therefore, it is essential to ensure consistent PPE compliance across all site personnel. Conventionally, monitoring PPE usage involves manual site inspections, physical patrols, or reviewing extensive surveillance footage. These traditional methods, however, are inefficient, labor-intensive, and prone to human error, particularly when inspectors experience fatigue from extended monitoring sessions^2^.

A promising alternative for detecting PPE non-compliance involves leveraging deep learning-based object detection on images sourced from surveillance systems or body-worn cameras. Object detection technology localizes and identifies specific objects within images by using bounding boxes, significantly automating tasks traditionally performed manually. Training such systems on extensive datasets, representing varied lighting conditions and object sizes, enables the deep learning models to generalize robustly and enhance detection reliability^3,4^. The You Only Look Once (YOLO) framework has become particularly prominent in the construction sector due to its rapid detection capabilities and ongoing enhancements across versions^5–7^.

This research employs YOLOv10 because of its enhanced robustness, accuracy improvements, and versatile adaptability compared to its predecessors. YOLOv10 exhibits superior performance in feature representation, handling diverse object scales, and effectively managing partial occlusions. Its modular architecture enables customization, particularly regarding the backbone network, crucially impacting both detection precision and processing speed by extracting essential features from input images^8^.

Conventionally, Convolutional Neural Network (CNN)-based backbones have dominated the field owing to their consistent performance^9,10^. However, recent developments in transformer-based backbones, including Vision Transformer (ViT), Swin Transformer, Pyramid Vision Transformer (PVT), MobileViT, and Axial Attention Transformer, have demonstrated significant potential in improving object detection across various domains^11,12^. To assess whether these contemporary architectures meet both the accuracy and real-time detection requirements essential for effective helmet compliance monitoring, this study evaluates multiple YOLOv10 configurations employing different transformer-based backbone models. The evaluation specifically focuses on detecting helmets in images captured from both stationary surveillance systems and mobile body-worn cameras. The primary contributions of this study are:

1. Utilizing datasets collected from construction site surveillance systems, body-worn camera footage, and benchmark datasets to comprehensively evaluate PPE non-compliance detection.
2. Proposing and customizing YOLOv10-based detection methods enhanced with advanced transformer architectures tailored specifically for detecting PPE items such as helmets, masks, vests, gloves, and shoes.
3. Assessing and benchmarking the generalizability of the proposed YOLOv10-based approach through comprehensive comparisons with other object detection methods.

## Literature review

### Existing studies for vision-based non-PPE monitoring

Over recent decades, extensive research has focused on developing automated approaches using deep learning—particularly CNNs—to detect safety helmets in construction environments. Fang et al.^13^ proposed a helmet detection model using Faster Region-Based Convolutional Neural Networks (Faster R-CNN), achieving accuracy rates ranging from 90.1 to 98.4% across diverse scenarios. However, this model’s processing time was approximately 0.2 s per image, limiting real-time applicability. Gu et al.^14^ improved upon Faster R-CNN by incorporating multi-scale training and increased anchor strategies, enhancing accuracy by approximately 7%; however, they did not explicitly report processing speed.

Considering the necessity for both high detection accuracy and swift processing, single-stage detectors have gained popularity. Yang et al.^15^ employed an image pyramid structure combined with YOLOv3 and DarkNet53 to capture multi-scale feature representations, achieving 95.13% accuracy at only 0.017 s per image, surpassing Single Shot Detection (SSD) and Faster R-CNN. Yan and Wang^16^ modified the YOLOv3 backbone, integrating densely connected convolutional networks to significantly enhance detection speed by 52% and accuracy by 5.7%, without compromising processing time. Shen et al.^17^ adopted a distinctive two-stage approach, initially using a single-stage face detector based on VGG16, followed by regression-based mapping and DenseNet-based classification for helmets. Nath^18^ combined YOLOv3 for worker detection with VGG16, ResNet, and Xception classifiers for helmets and vests, achieving an accuracy of 71.23%. Wang et al.^19^ compared YOLOv5x and YOLOv5s, identifying YOLOv5x as the more accurate option (86.55%) and YOLOv5s as faster (0.019 s per frame). Lee et al.^20^ introduced a real-time detection framework based on You Only Look At CoefficienTs (YOLACT) with MobileNetV3, achieving 66.4% accuracy for helmets and vests. Nguyen et al.^21^ improved YOLOv5 models using the Seahorse Optimization algorithm (SHO), achieving 66% average accuracy across multiple PPE categories (helmets, masks, gloves, vests, shoes). Park et al.^22^ highlighted the advantages of individual augmentation strategies using YOLOv8 with transformer architectures such as Swin Transformer and Axial Transformer to enhance non-PPE detection performance.

The existing literature is summarized in Table 1. These studies collectively highlight the effectiveness of various deep learning techniques for real-time PPE compliance monitoring. However, direct comparisons among these studies remain challenging due to their reliance on diverse, often non-public datasets. Moreover, due to unreported hyperparameter optimizations and omitted architectural details, exact replication of existing methods is challenging; thus, this research implements methods from existing studies as closely as possible to facilitate a fair comparative analysis.

### Transformer-based architectures for YOLOv10

In YOLO-based object detection frameworks, the choice of backbone architecture significantly influences the system’s capability to accurately detect and classify objects through effective extraction of meaningful image features^6^. Recently, transformer-based backbone architectures have garnered considerable attention due to their distinct advantages over traditional CNN-based methods. CNN backbones primarily utilize convolutional layers optimized for capturing localized spatial features, which, although efficient for real-time detection, tend to lack extensive global contextual awareness, limiting their effectiveness in complex scenarios or detecting smaller-scale objects^23^.

Transformer-based backbones, by contrast, leverage self-attention mechanisms to capture extensive local and global context simultaneously. This enhanced contextual comprehension significantly improves detection accuracy, especially within visually complex environments common on construction sites^24^. However, previous integrations of transformer architectures, such as combinations of YOLOv8 with Swin or Axial Transformers, often encountered increased computational demands or suboptimal implementations, thus constraining real-time applicability.

The challenge of accurately monitoring multiple PPE items simultaneously within complex images under strict real-time constraints highlights a critical research gap. As summarized in Table 1, existing detection methods exhibit potential limitations compared to the proposed YOLOv10 approach. Specifically, two-stage methods such as Faster R-CNN are inherently slower due to separate region proposal and classification processes, resulting in lower inference speeds. Traditional CNN-based YOLO methods, while faster, often struggle with accurately detecting small or challenging objects due to limited global context awareness. Methods involving additional classifiers after YOLO detection, such as VGG16, ResNet, and Xception, further reduce real-time performance due to multi-step processes. Additionally, instance segmentation methods like Mask R-CNN have inherently higher computational costs, resulting in slower inference^25,26^. Optimization techniques like SHO on YOLOv5, though effective, provide moderate accuracy improvements but still exhibit limitations in robustness to diverse object scales and complexities.

This study systematically evaluates and compares several advanced transformer-based backbone architectures—ViT, Swin Transformer, and PVT—within the YOLOv10 detection framework, specifically targeting automated non-PPE detection. The primary objective is to achieve an optimal balance between accuracy and real-time efficiency under realistic construction site conditions.

**Table 1:** Summary of existing literature for non-PPE detection.

Reference	Target PPE	Dataset	Method	Potential limitation compared to proposed method
Fang et al.^13^	Helmet	Custom dataset (publicly not open)	Faster R-CNN	Lower inference speed and detection accuracy
Gu et al.^14^	Helmet	Custom dataset (publicly not open)	Faster R-CNN with multi-scale training	Lower generalization, limited small-object detection
Yang et al.^15^	Helmet	Custom dataset (publicly not open)	YOLOv3 with DarkNet53	Inferior accuracy and weaker performance on small objects
Yan and Wang^16^	Helmet	Custom dataset (publicly not open)	YOLOv3 with DarkNet53	Less robust detection performance and slower inference
Shen et al.^17^	Helmet	Custom dataset (publicly not open)	VGG16-based face detector (Face); DenseNet-based classifier (Helmet)	Two-stage method, slower inference, and limited scalability
Nath^18^	Helmet, and vest	Custom dataset (publicly not open)	YOLOv3 (worker detection); VGG16, ResNet, Xception (helmet and vest classifiers)	Two-stage classification, lower real-time detection capability
Wang et al.^19^	Helmet, and vest	Custom dataset (publicly not open)	YOLOv5x and YOLOv5s	Lower AP, reduced accuracy on challenging small-scale objects
Lee et al.^20^	Helmet, and vest	Custom dataset (publicly not open)	Mask R-CNN with MobileNetV3	Slower inference speed due to complex instance segmentation
Nguyen et al.^21^	Helmet, mask, glove, vest, shoes	Custom dataset (publicly not open)	SHO-based YOLOv5	Moderate detection accuracy and limitations in object scale robustness
Park et al.^22^	Helmet, mask, glove, vest, shoes	Custom dataset (publicly not open)	YOLOv8, Swin Transformer, Axial Transformer	Slightly slower inference speed and lower overall accuracy

## Methods

The following sections present detailed descriptions of the various YOLOv10-based models and their differing architectures, followed by a comprehensive overview of the overall methodology.

### YOLOv10 framework

Figure 2 illustrates the workflow of the proposed YOLOv10-based detection approach, consisting of three primary components: a backbone, a neck, and a head. The backbone extracts multi-scale features from the input images, effectively capturing both low-level image details and higher-level semantic content. The neck employs a Path Aggregation Network (PAN), which integrates these extracted features across multiple scales through interconnected pathways^27^.

**Fig. 1:**
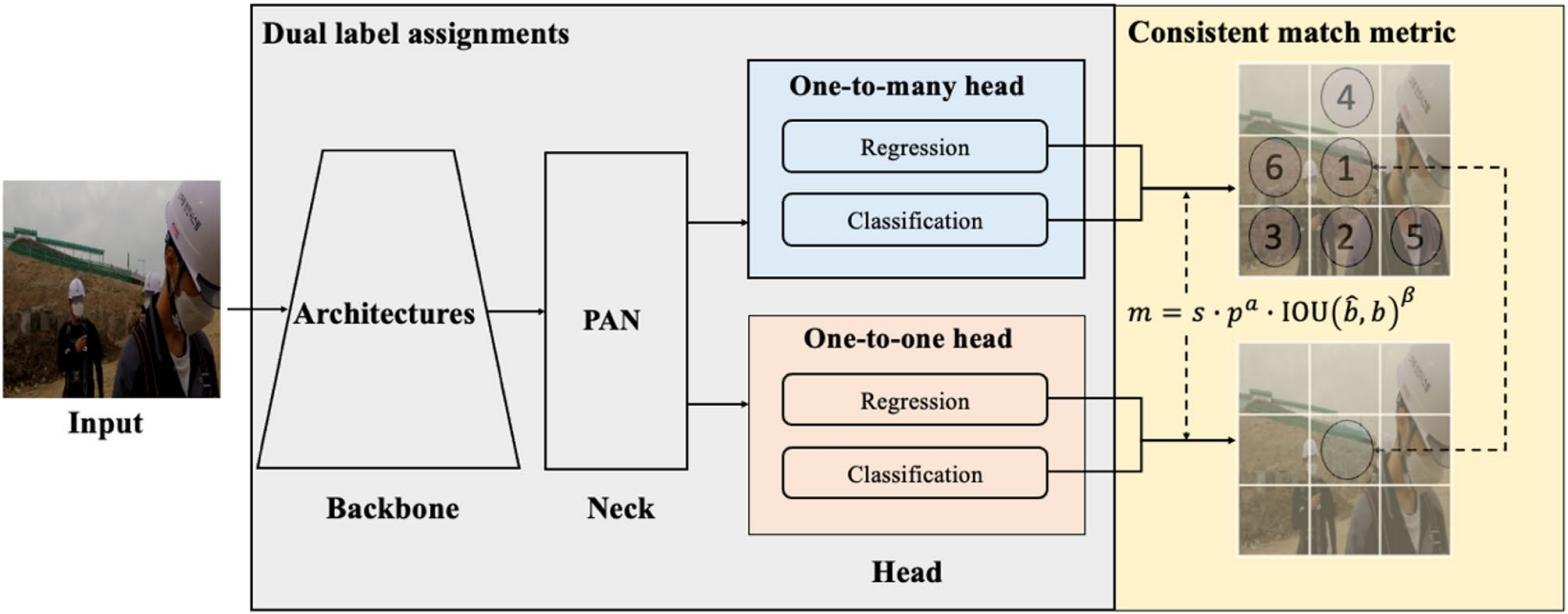
Workflow of YOLOv10-based method for detecting non-PPE.

The head incorporates a dual-label assignment strategy: firstly, a one-to-many mechanism generates multiple candidate predictions per object; secondly, a one-to-one mechanism uniquely pairs each prediction to the corresponding ground-truth bounding box. A consistent matching metric combining confidence scores, classification probabilities, and Intersection over Union (IOU) with ground-truth boxes is applied:

$$\:m=s\bullet\:{p}^{a}\bullet\:\text{I}\text{O}\text{U}{\left(\widehat{b},\:b\right)}^{\beta\:}$$

In this equation, $\:s$ represents the prediction confidence, $\:{p}^{a}$ denotes the classification probability raised to the exponent $\:a$, $\:\text{I}\text{O}\text{U}{\left(\widehat{b},\:b\right)}^{\beta\:}$ calculates the overlap between the predicted bounding box $\:\widehat{b}$ and the actual ground-truth bounding box $\:b$. The adjustable parameters $\:a$, and $\:\beta\:$ regulate the relative contributions of these factors. Employing this matching strategy enhances YOLOv10’s accuracy and consistency in predictions, effectively addressing challenges posed by varying object boundaries across diverse detection scenarios.

### Backbone architectures

The sections below summarize key aspects of each architecture. For comprehensive explanations and technical analysis of these architectures, refer to Khan et al.^28^.

#### ViT

**Fig. 2:**
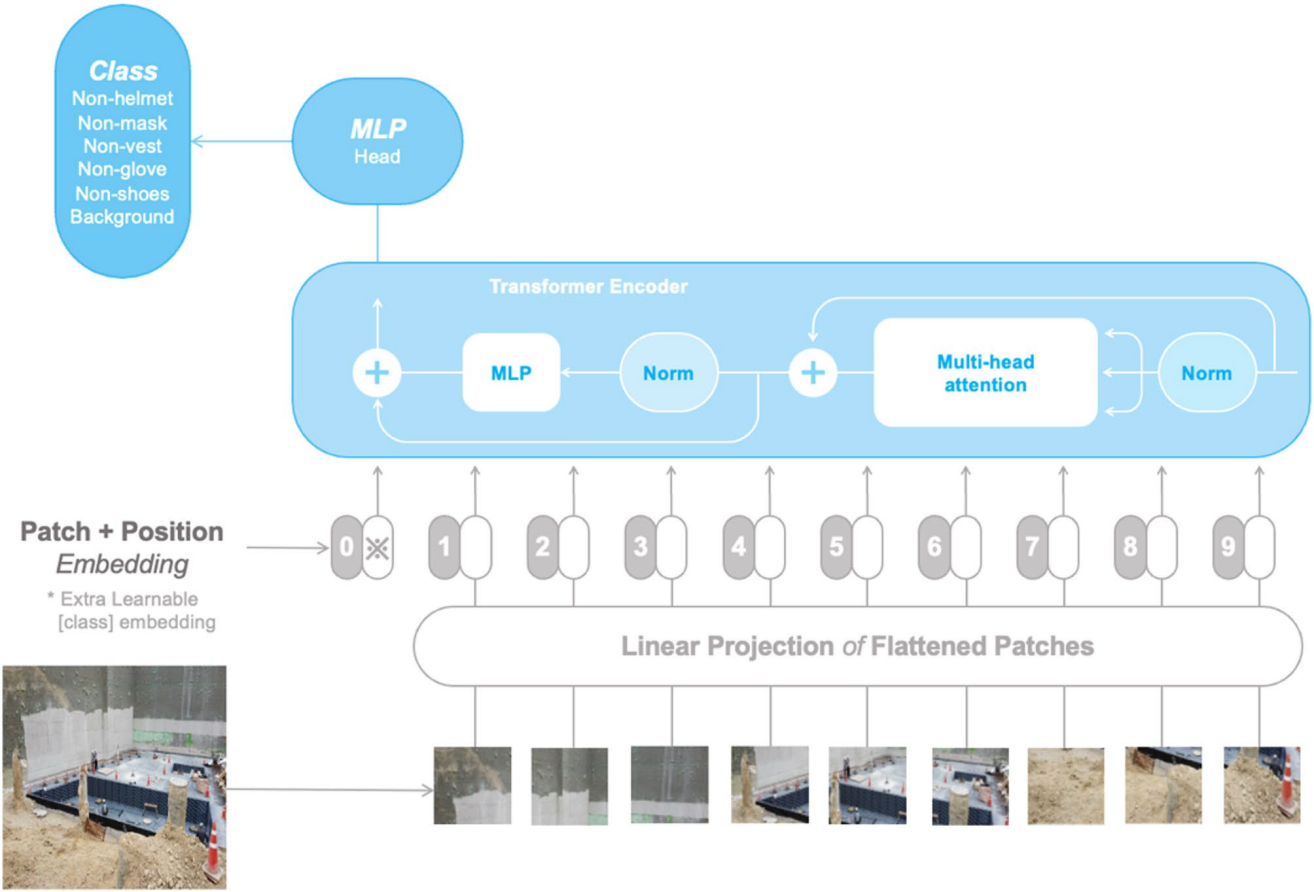
Architecture of ViT for non-PPE detection.

ViT is a purely transformer-based architecture designed for visual recognition tasks. It operates by segmenting the input images into fixed-size patches, treating each as an independent token (Fig. 2). The principal advantage of ViT is its capability to apply self-attention mechanisms directly to these tokens, allowing it to effectively capture global dependencies within images. However, ViT generally requires large-scale datasets and significant computational resources, as the complexity of its self-attention mechanisms scales quadratically with the number of patches^2^.

#### Swin transformer

**Fig. 3:**
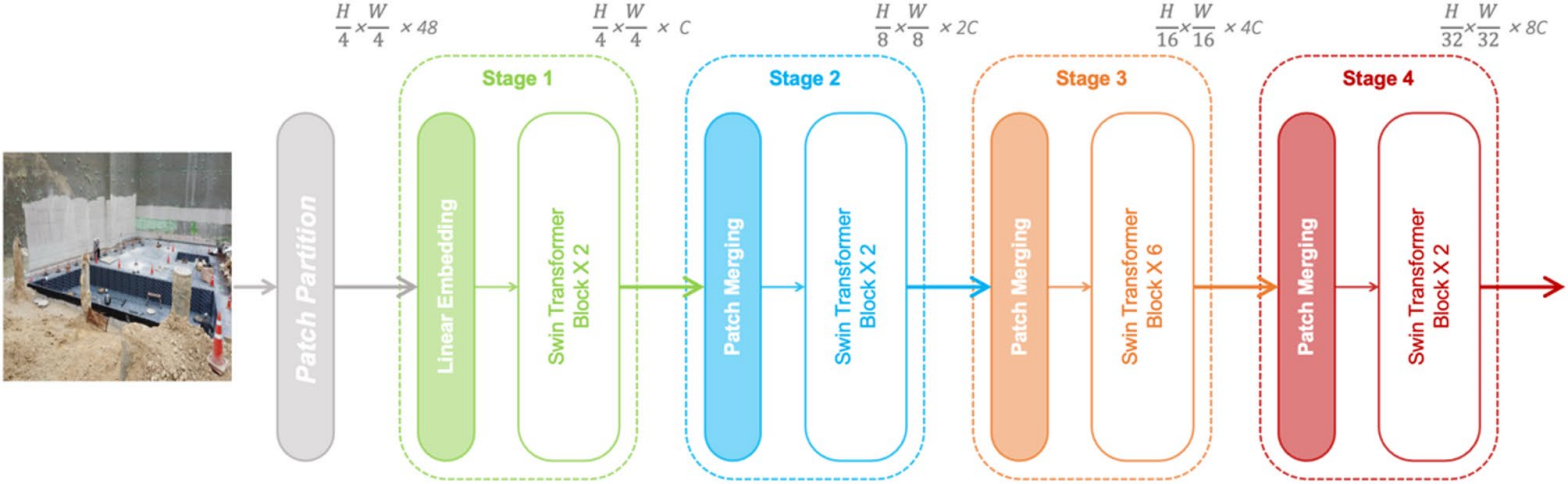
Architecture of Swin Transformer for non-PPE detection.

Swin Transformer employs a hierarchical architecture featuring shifted-window self-attention mechanisms (Fig. 3). Initially, the input image is divided into local windows without overlap, after which these windows are shifted between layers to capture interactions across adjacent regions. This method results in linear complexity regarding attention operations relative to image size. Additionally, its hierarchical structure facilitates multi-scale feature extraction, effectively capturing both detailed local features and broader contextual information^29^.

#### PVT

**Fig. 4:**
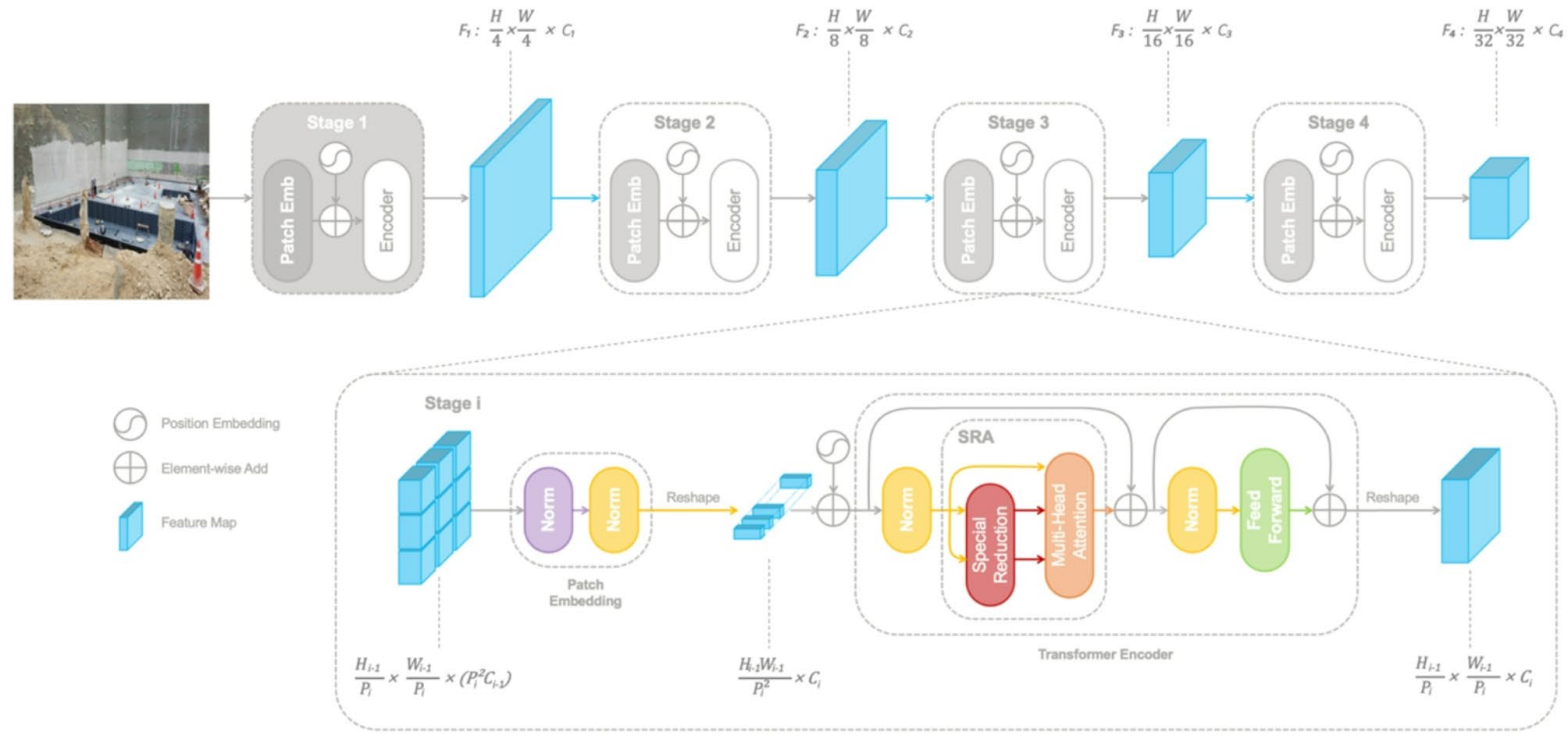
Architecture of PVT for non-PPE detection.

PVT combines pyramid-based multi-scale feature extraction—traditionally used by CNNs—with a transformer-based framework (Fig. 4). It employs a spatial-reduction attention mechanism that reduces the spatial dimension within attention layers to efficiently generate multi-scale feature representations. This pyramid structure maintains the global contextual advantages associated with transformer architectures while significantly improving computational efficiency, effectively balancing high accuracy with reduced computational demands^30^.

### Optimization process

#### Transfer learning

Training a deep learning model from scratch allows for greater flexibility and customization but requires significant computational resources and a large dataset. A practical and widely-used alternative is employing a pre-trained model, which has already learned generalizable features from extensive datasets and can transfer this knowledge to new, related tasks efficiently^31,32^. However, pre-trained models are often trained on datasets within specific contexts, potentially introducing biases or domain mismatches when applied to significantly different domains. This mismatch could lead to reduced performance if the new task’s data substantially differs from the original training set^33^.

In this study, ImageNet^34^ was selected for pre-training the feature extraction layers of our chosen model architectures. With over 14 million images covering diverse categories such as vehicles and people, ImageNet provides a robust and generalizable foundation.

#### Hyperparameter

Training deep learning models for high accuracy typically requires careful optimization of multiple hyperparameters, as model performance can vary significantly based on their configurations^35,36^. Although no universal hyperparameter set exists that guarantees optimal results across all tasks and datasets, a common and practical strategy involves adopting hyperparameter choices informed by existing literature and empirical evidence^37^. Hyperparameters proven effective in closely related tasks frequently yield strong results in similar contexts. Consequently, this research selected hyperparameter values guided by successful implementations of YOLO-based methods, such as those used in rebar counting applications on construction sites^8^.

### Evaluation of model performance

#### AP

In object detection tasks, Average Precision (AP) is widely adopted as a standard evaluation measure to assess model effectiveness^38^. AP summarizes precision at various recall levels across a range of Intersection over Union (IOU) thresholds. IOU evaluates how accurately the predicted bounding boxes overlap with the ground-truth objects.

In this study, detection accuracy is evaluated using two primary AP metrics: AP50 and AP50:95. Specifically, AP50 denotes the precision calculated at an IOU threshold of 0.5, while AP50:95 represents the averaged precision over multiple IOU thresholds ranging from 0.5 to 0.95, providing insights into model robustness across different localization criteria.

Additionally, the performance of the detection model is analyzed based on the size of non-PPE objects detected. These are categorized according to pixel dimensions as follows:

- Small objects (AP_S): objects smaller than 32 × 32 pixels.
- Medium objects (AP_M): objects between 32 × 32 and 96 × 96 pixels.
- Large objects (AP_L): objects larger than 96 × 96 pixels.

For practical non-PPE monitoring scenarios, the AP50 metric is especially relevant, as operational priorities typically emphasize correctly identifying non-PPE items rather than achieving perfect bounding box alignment.

#### Detection speed

Detection speed indicates how rapidly the model can process and analyze each image, commonly expressed as frames per second (FPS). Evaluating detection speed is essential to confirm whether the YOLO-based model meets the real-time processing requirements essential for practical non-PPE monitoring applications.

## Experiment

### Data Preparation

#### Original dataset construction

In this research, data was gathered from two main sources: on-site data collection and publicly accessible datasets. On-site data collection involved using two camera setups across several construction sites in South Korea. Specifically, seven sites were monitored using 42 strategically placed Bosch FLEXIDOME IP Starlight 8000i surveillance cameras to provide comprehensive visual coverage. Additionally, GoPro HERO9 Black cameras were attached to safety helmets worn by 35 construction workers at five of these sites. Due to financial constraints, body-worn cameras were not implemented at the remaining two sites.

**Fig. 5:**
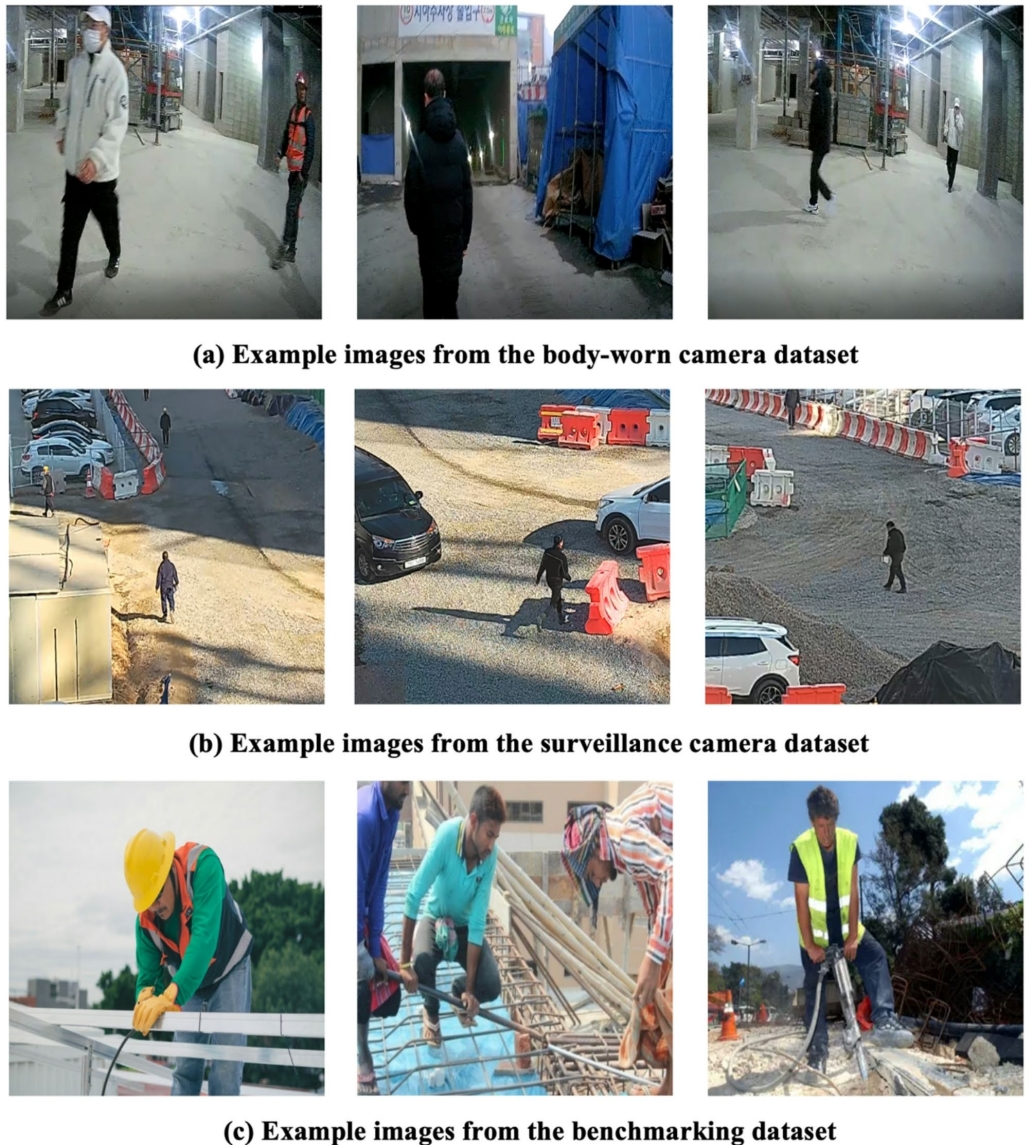
Example images from each source dataset.

Continuous video footage was captured, from which frames were systematically extracted at regular intervals to authentically represent on-site conditions. Frames obtained from the body-worn cameras and surveillance cameras are illustrated in Fig. 5(a) and (b), respectively. Initially, this extraction produced a large dataset of 85,875 images. To ensure balanced representation between scenarios—workers fully compliant with PPE requirements and those missing PPE—a subset of 2,297 representative images was selected.

To further enrich the dataset, publicly available resources were incorporated, specifically the Safety Helmet Wearing Dataset (SHWD)^39^ consisting of 530 images and the Safety Helmet Computer Vision Project (SHCVP)^40^ containing 565 images. These additional images, exemplified in Fig. 5(c), expanded the visual diversity and context coverage for non-PPE detection tasks. In total, 3,392 images were consolidated from these combined sources.

#### Data split

The collected dataset was randomly partitioned into three subsets: training, validation, and testing, using proportions of 60%, 20%, and 20%, respectively. Specifically, from the complete dataset of 3,392 images, 2,036 images were allocated for training, 678 images for validation, and 678 images for testing. The dataset acquired from body-worn cameras was similarly divided according to the same proportional distribution. This method ensures an unbiased and thorough evaluation, thereby facilitating a reliable assessment of the developed detection models.

#### Image augmentation techniques

**Fig. 6:**
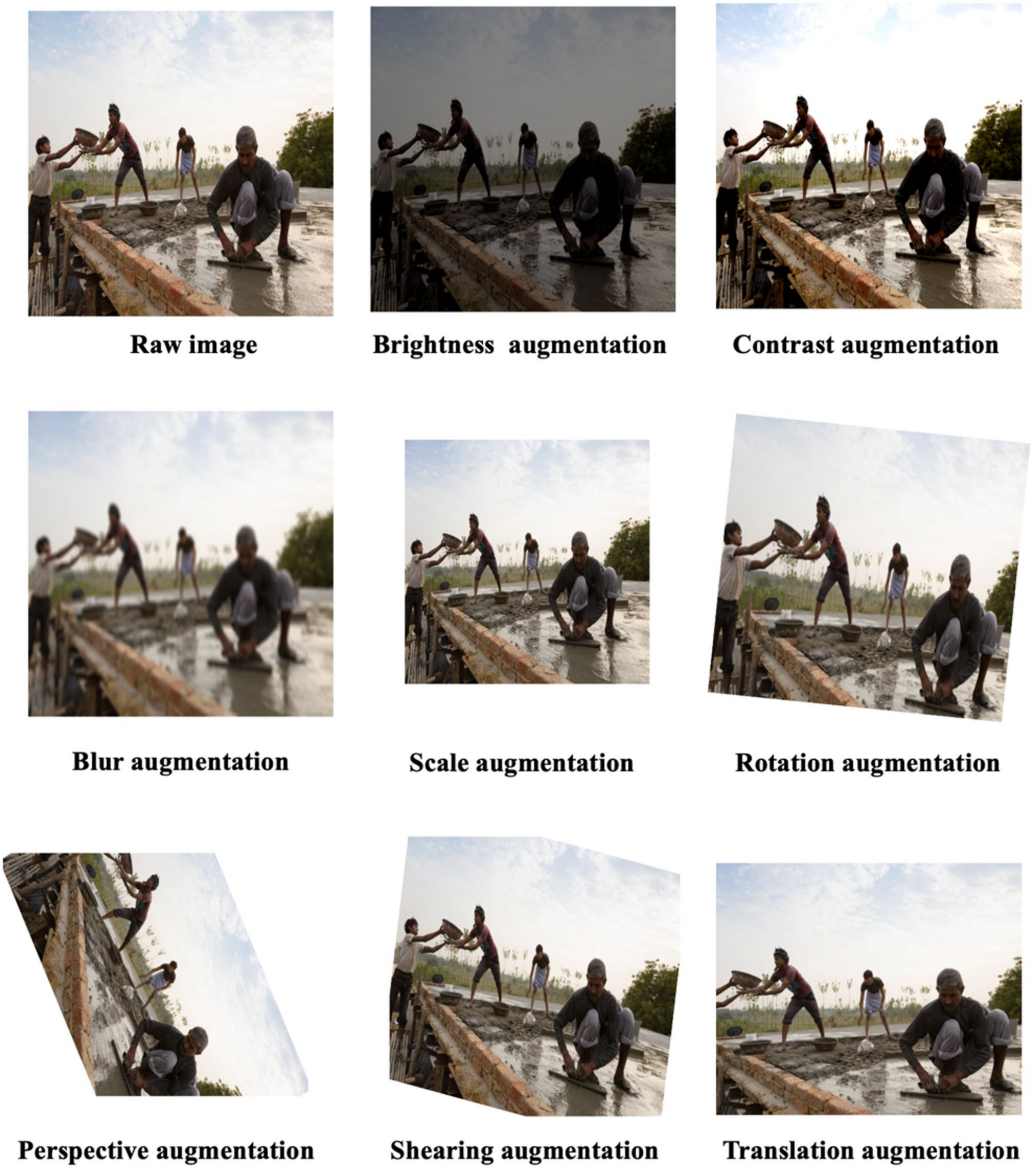
Examples of images augmentation techniques.

Image augmentation parameters significantly affect both image quality and the resulting performance of detection models. Preliminary analyses indicated stricter detection requirements for safety helmets compared to other PPE categories. Consequently, augmentation methods were selectively applied to a targeted subset of 102 images featuring non-safety-helmet scenarios. Eight distinct augmentation techniques were used to artificially enlarge this dataset, resulting in a total of 816 augmented images. These techniques and their parameters were carefully selected through iterative experimentation to produce realistic augmentations, summarized in Table 2, and visually demonstrated in Fig. 6.

**Table 2:** Applied augmentation techniques with their respective parameters and values.

Augmentation techniques	Parameters	Ranges of values
Brightness	β	[−50, 50]
Contrast	α	[0.3, 2.7]
Blurring	$\:\theta\:$	[0, 1]
Scale	α	[0.5, 2.0]
Rotation	$\:{a}_{31}$,​$\:{a}_{32}$​,	[0.01, 0.15]
Perspective	x, y	[0.8, 1.2], [0.8, 1.2]
Shearing	$\:\theta\:$	[− 25°, 25°]
Translation	$\:\theta\:$	[0, 1]

#### Annotation

**Fig. 7:**
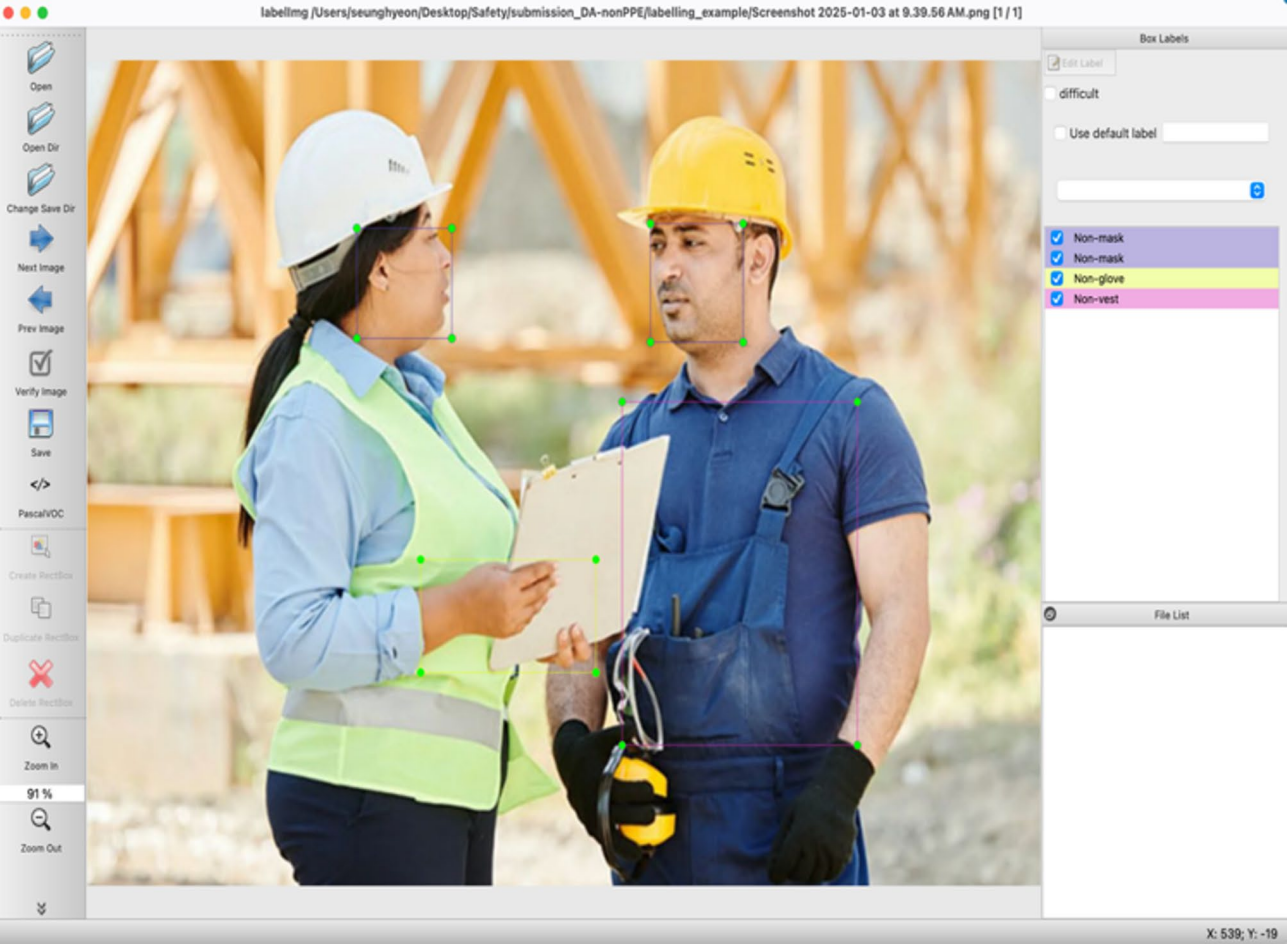
Examples of annotation process for detecting non-PPE.

For annotation purposes, each occurrence of missing PPE was identified by placing rectangular bounding boxes around five distinct categories: “Non-helmet,” “Non-mask,” “Non-glove,” “Non-vest,” and “Non-shoes.” All other areas not associated with these categories were labeled as “background.” The annotation process was carried out using LabelImg, generating annotations in the VOC XML format. An illustrative example of this annotation process is provided in Fig. 7.

#### Synthesis of final dataset

To evaluate the proposed methodology, datasets from both construction-specific and general contexts were collected and subsequently divided into training, validation, and testing subsets. Before labeling, eight augmentation techniques were selectively applied only to images containing non-safety helmet scenarios, addressing class imbalance caused by their initially limited number. Each image was then labeled according to specific non-PPE categories. Table 3 summarizes the annotated datasets, clearly outlining the distribution of each non-PPE category within the respective subsets.

**Table 3:** Comprehensive dataset distribution.

Purpose	Number of images	Class					
		Non-helmet	Non-mask	Non-glove	Non-vest	Non-shoes	Total
Training	2,036	356	1,463	852	836	921	4,428
Augmentation	816	2,848	221	326	117	221	3,733
Validation	678	121	425	327	399	378	1,650
Test	678	102	418	383	387	345	1,635
Total	4,208	3,427	2,527	1,888	1,739	1,865	11,446

### Experimental settings

All experiments were conducted on a workstation running Windows 10. The system featured a high-performance Intel Core i7-13700 H CPU equipped with 14 cores and 20 threads, along with 128 GB of RAM. Computational tasks were accelerated using a powerful NVIDIA GeForce GTX 4090 Ti GPU.

## Results, and discussion

### Results of training and validation

#### Assessment of underfitting, and overfitting

**Fig. 8:**
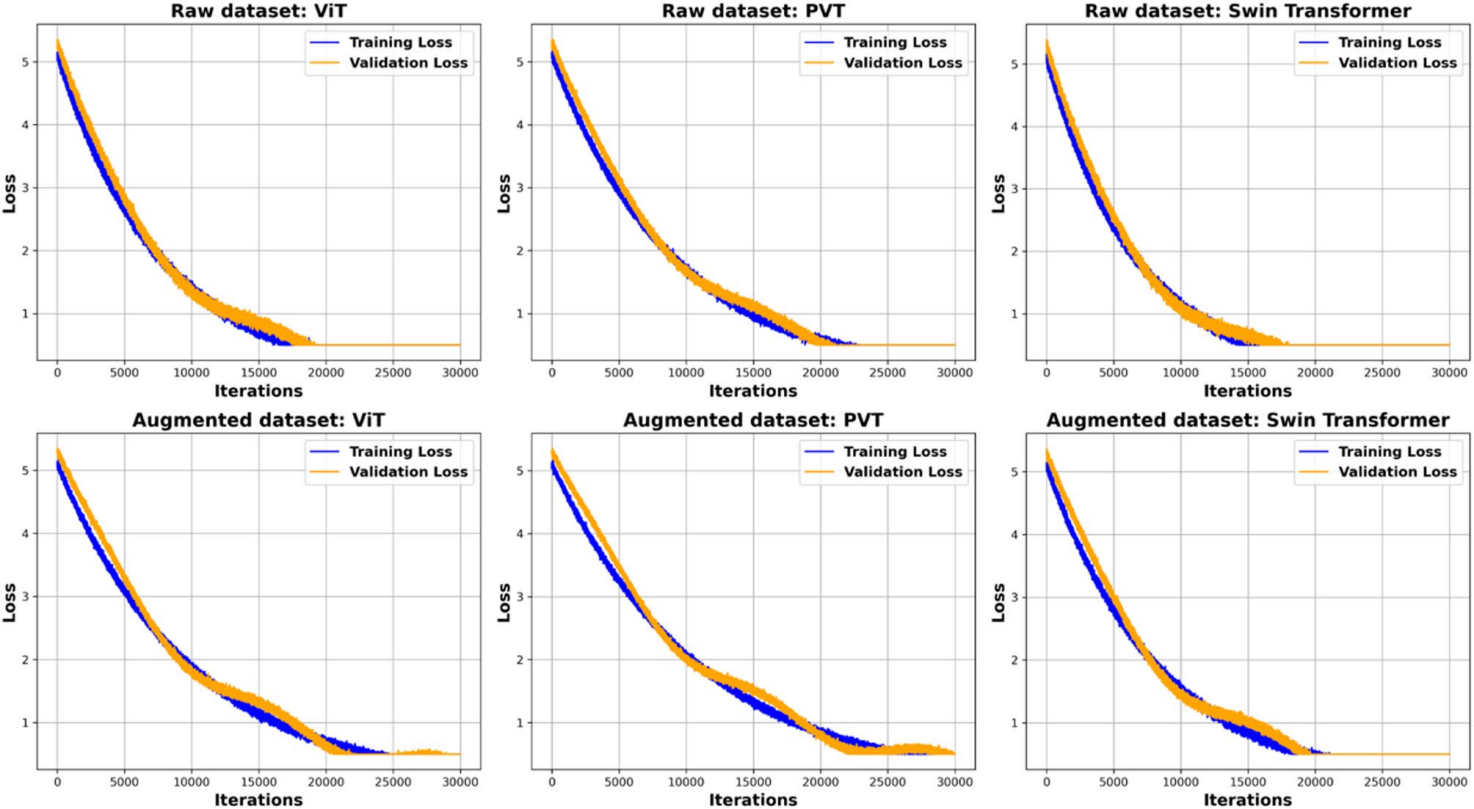
Training and validation loss curves.

Figure 8 presents the training and validation loss curves for three YOLOv10 transformer-based models, each employing a distinct backbone architecture, evaluated on both raw and augmented datasets at 30,000 iterations. Throughout the training process, all models demonstrated a steady decline in loss for both the training and validation sets, although slight fluctuations occurred across epochs. This steady reduction indicates successful model training and improved generalization on the validation data, ultimately nearing convergence. The continuous decrease in the training loss further confirms the absence of underfitting, whereas the consistent trend and small gap between training and validation losses suggest that overfitting was not problematic. Consequently, none of the evaluated models required exclusion due to concerns related to underfitting or overfitting.

#### Analysis of augmentation techniques effectiveness in overall performance

As shown in Table 4, all three architectures (ViT, Swin Transformer, and PVT) notably benefited from data augmentation. For AP50, ViT demonstrated the highest improvement (+ 3.36%), closely followed by PVT (+ 3.05%) and Swin Transformer (+ 2.97%). For stricter localization (AP50:95), ViT again exhibited the largest gain (+ 5.28%), followed by Swin Transformer (+ 4.74%) and PVT (+ 3.95%).

Improvements in IOU were highest for Swin Transformer (+ 5.41%), suggesting that its hierarchical attention mechanism effectively adapted to augmentation-induced transformations. Gains were particularly pronounced for detecting small objects (AP_S), with Swin Transformer achieving the highest improvement (+ 7.54%), closely followed by ViT (+ 7.14%) and PVT (+ 5.61%). Medium (AP_M) and large (AP_L) objects showed smaller but still meaningful improvements, consistently favoring Swin Transformer.

**Table 4:** Comparison results of model performance across different architectures under augmentation.

Metrics	ViT			Swin Transformer			PVT			
	Raw	Aug	Gain	Raw	Aug	Gain	Raw	Aug	Gain	
AP50	83.07	86.43	3.36	84.28	87.25	2.97	83.4	86.45	3.05	
AP50:95	58.53	63.81	5.28	59.78	64.52	4.74	58.92	62.87	3.95	
IOU	76.22	81.41	5.19	77.15	82.56	5.41	76.58	80.74	4.16	
$\:{AP}_{S}$	52.21	59.35	7.14	53.4	60.94	7.54	53.15	58.76	5.61	
$\:{AP}_{M}$	80.13	83.42	3.29	80.41	84.93	4.52	80.22	83.33	3.11	
$\:{AP}_{L}$	87.02	90.12	3.1	88.23	91.59	3.36	87.47	89.91	2.44	

#### Analysis of augmented model performance with different architectures by class

The Swin Transformer consistently achieved the highest overall performance, with average AP50 (87.25), AP50:95 (64.52), and IOU (82.56), surpassing both ViT and PVT, as presented in Table 5. Among individual classes, non-helmet detection achieved the highest accuracy (AP50 > 91%), indicating distinctive visual characteristics. Conversely, detection accuracy was lowest for non-glove and non-shoes (approximately 82–85% AP50), likely due to visual ambiguity or occlusion issues. Performance consistently increased with object size (AP_S, AP_M, AP_L), and Swin Transformer notably achieved the best results across these metrics, highlighting its effectiveness in capturing detailed features, especially for smaller or partially occluded items.

**Table 5:** Class-wise performance of augmented models using different architectures.

Architecture	Metric	Class					
		Non-helmet	Non-mask	Non-vest	Non-glove	Non-shoes	mAP
ViT	AP50	91.48	87.13	86.26	84.51	82.77	86.43
	AP50:95	67.54	64.32	63.68	62.39	61.11	63.81
	IOU	86.17	82.07	81.25	79.6	77.96	81.41
	AP_S	62.82	59.83	59.23	58.03	56.84	59.35
	AP_M	88.3	84.09	83.25	81.57	79.89	83.42
	AP_L	95.39	90.85	89.94	88.12	86.3	90.12
Swin Transformer	AP50	92.35	87.95	87.07	85.32	83.56	87.25
	AP50:95	68.29	65.04	64.39	63.09	61.79	64.52
	IOU	87.39	83.23	82.39	80.73	79.06	82.56
	AP_S	64.5	61.43	60.82	59.59	58.36	60.94
	AP_M	89.9	85.61	84.76	83.05	81.33	84.93
	AP_L	96.95	92.33	91.41	89.56	87.71	91.59
PVT	AP50	91.5	87.15	86.28	84.53	82.79	86.45
	AP50:95	66.55	63.38	62.74	61.48	60.21	62.87
	IOU	85.46	81.39	80.58	78.95	77.32	80.74
	AP_S	62.2	59.23	58.64	57.46	56.27	58.76
	AP_M	88.2	83.95	83.13	81.48	79.8	83.33
	AP_L	95.17	90.64	89.73	87.92	86.1	89.91

Table 6 indicates that PVT achieved the fastest inference speed (36.54 FPS), closely followed by Swin Transformer (35.32 FPS) and ViT (34.26 FPS). All models demonstrated consistent and stable inference times, exhibiting minimal variability across different conditions, as indicated by narrow ranges between minimum and maximum FPS values. Overall, Swin Transformer delivered superior detection accuracy across various classes and object sizes, while PVT provided the highest inference speed, making these architectures suitable for high-precision and real-time applications, respectively, in construction-site environments. Additionally, the detailed Floating Point Operations (FLOPs)—a measure of computational complexity indicating the number of floating-point calculations required—and parameter counts (M) for each method are summarized in Table 7.

**Table 6:** Summary of inference speed (FPS) by architectures.

Architecture	Statistics					
	Mean	Min	25%	50%	75%	Max
ViT	34.26	34.21	34.37	34.47	34.82	35.23
Swin Transformer	35.32	34.78	34.56	35.26	35.67	36.35
PVT	36.54	34.16	35.43	35.55	36.74	37.13

**Table 7:** FLOPs and parameter counts in each method.

Detector	Architecture	FLOPs (G)	Parameters (M)
Faster R-CNN	ResNet-101	253.7	61.2
	ResNet-152	302.5	76.8
	MobileNetv3	72.4	14.9
SSD500	ResNet-101	149.8	34.7
	ResNet-152	171.3	48.5
	MobileNetv3	49.6	10.1
R-FCN	ResNet-101	188.7	42.2
	ResNet-152	222.6	56.9
	MobileNetv3	59.8	11.3
YOLOv5	CSPDarknet53s	35.8	7.3
	CSPDarknet53x	54.2	18.4
YOLOv8	Swin Transformer	64.7	23.6
	PVT	60.2	20.4
	Axial Transformer	57.9	19.7
YOLOv10	CSPNet	61.7	21.8
	ConvNeXt	59.4	23.5
	EfficientNet	45.3	16.2
	ViT	84.6	28.9
	Swin Transformer	67.8	24.1
	PVT	65.9	23.2

### Model evaluation in test images

#### Overall performance

**Fig. 9:**
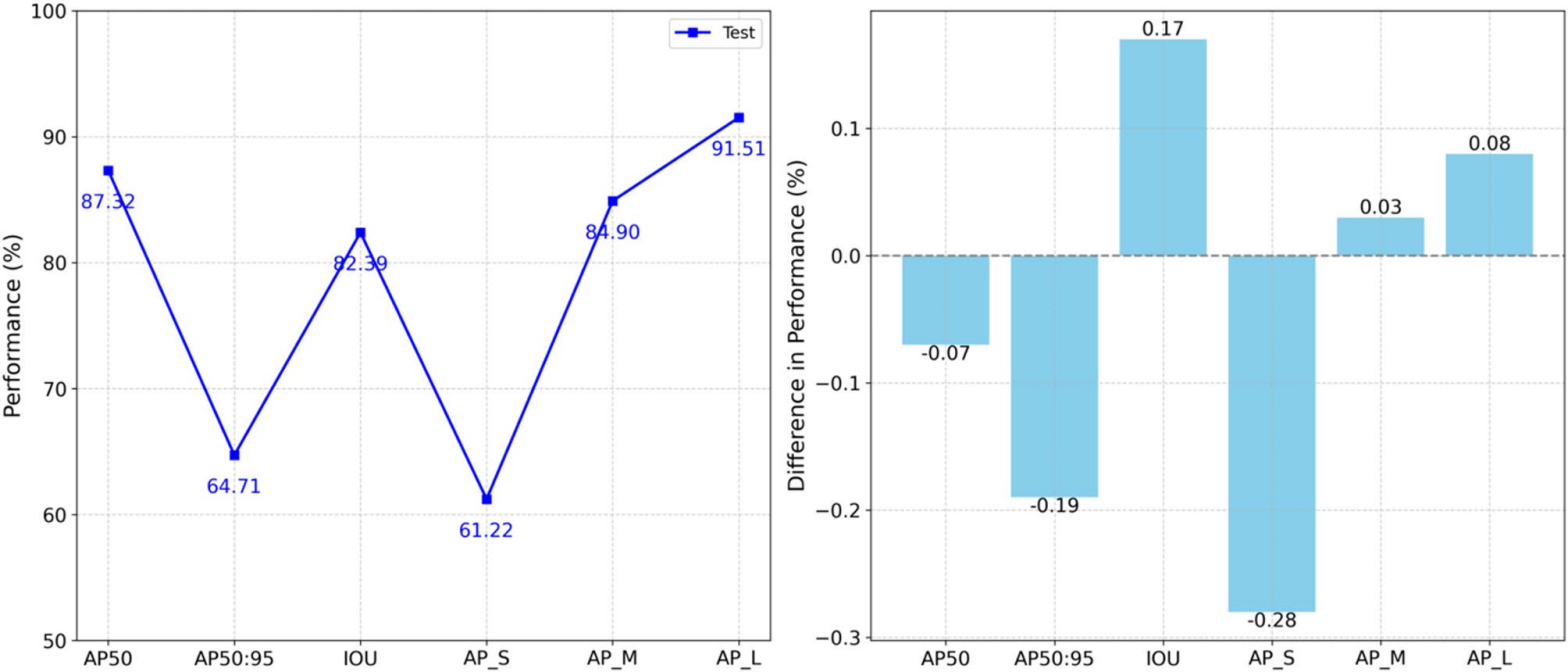
Best model performance on test data (Swin Transformer).

The best-performing Swin Transformer model was evaluated using previously unseen test data, demonstrating excellent consistency with validation results. This consistency indicated minimal overfitting and robust generalizability, as summarized in Fig. 9. Table 8 further details the class-wise performance metrics. Detection performance varied significantly among classes. Categories with visually distinctive features, such as non-helmet, achieved high accuracy (exceeding 92%). In contrast, visually challenging categories, such as non-shoes, had comparatively lower accuracy (~ 83.5%), likely due to practical difficulties such as occlusions and visual ambiguity.

**Fig. 10:**
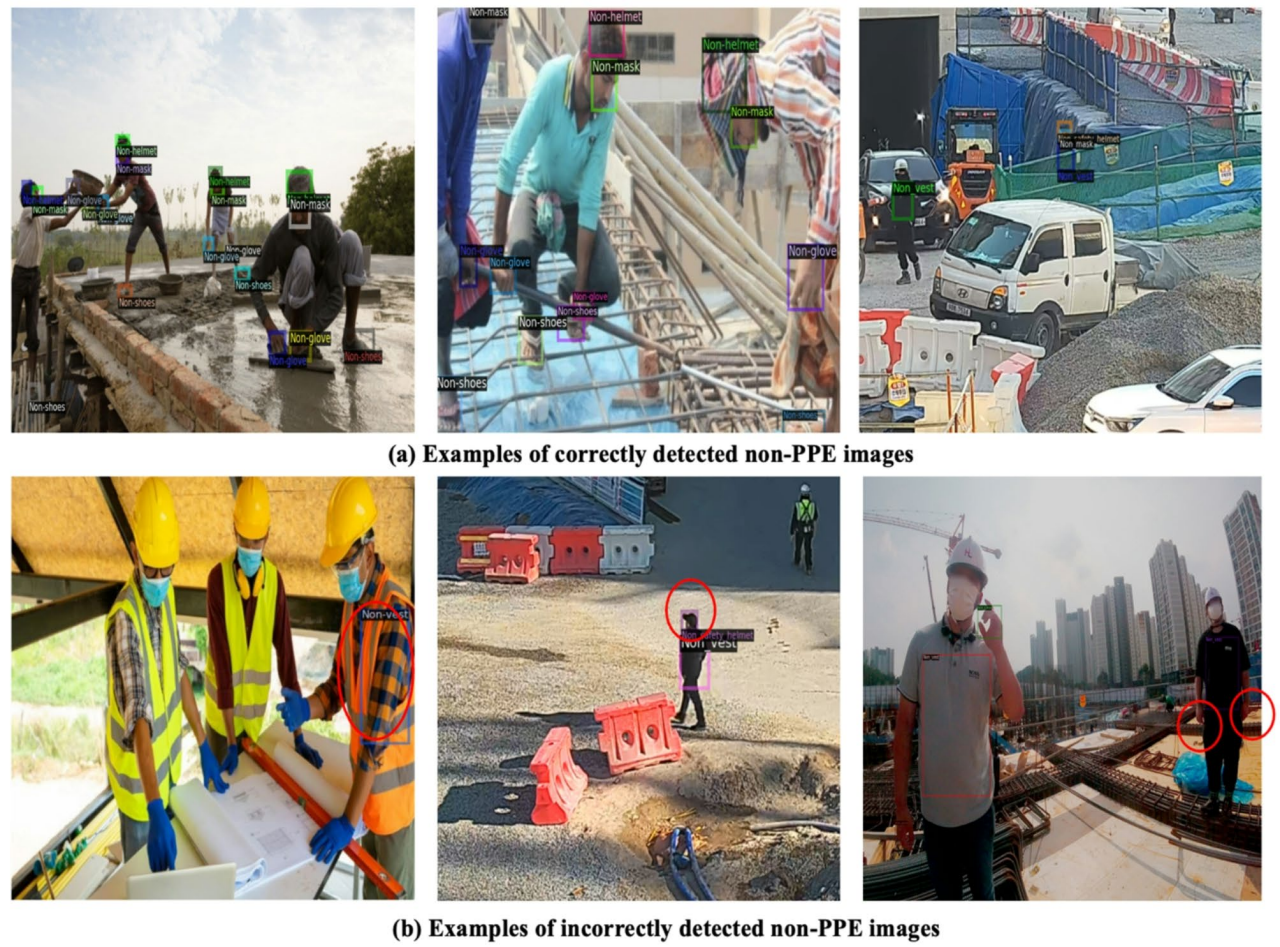
Visual examples of correctly, and incorrectly detected images.

Figure 10 provides visual examples of correctly and incorrectly detected non-PPE objects. The model demonstrated notably better performance in detecting larger objects compared to smaller ones, highlighting the effectiveness of the Swin Transformer’s hierarchical attention mechanism in addressing multi-scale detection challenges. However, there was a noticeable decrease in performance under stricter localization thresholds (AP50:95 approximately 64.5%), emphasizing the inherent difficulties in precisely aligning bounding boxes in complex real-world construction scenarios.

**Table 8:** Class-wise results of validation and test.

Datasets	Metrics	Class					
		Non-helmet	Non-mask	Non-vest	Non-glove	Non-shoes	mAP
Validation	AP50	92.35	87.95	87.07	85.32	83.56	87.25
	AP50:95	68.29	65.04	64.39	63.09	61.79	64.52
	IOU	87.39	83.23	82.39	80.73	79.06	82.56
	AP_S	64.5	61.43	60.82	59.59	58.36	60.94
	AP_M	89.9	85.61	84.76	83.05	81.33	84.93
	AP_L	96.95	92.33	91.41	89.56	87.71	91.59
Test	AP50	92.4	88.17	87.17	85.36	83.48	87.32
	AP50:95	68.23	65.43	64.85	62.97	62.08	64.71
	IOU	87.46	83.66	81.93	80.32	78.58	82.39
	AP_S	64.78	61.8	61.3	59.89	58.32	61.22
	AP_M	89.52	85.75	84.4	83.49	81.35	84.9
	AP_L	96.71	92.6	91.37	89.63	87.23	91.51

#### Impact of image conditions

Table 9 summarizes the model’s detection performance (AP50) across various image conditions. The analysis reveals that detection accuracy consistently decreased under more complex scenarios, notably low illumination, far-field views, outdoor environments, overlapping workers, grouped workers, and scenarios involving multiple individuals. Among these factors, illumination and visual range exerted the greatest influence on detection accuracy, with high-illumination conditions and near-field views achieving noticeably higher mean average precision (mAP: approximately 88.2%) compared to low illumination and far-field conditions (mAP around 86.4%).

**Table 9:** Results of the best model, grouped by different image conditions at AP50 level.

Image conditions	Ranges	Class					
		Non-helmet	Non-mask	Non-vest	Non-glove	Non-shoes	mAP
Illumination	High	92.91	89.49	88.27	86.44	84	88.22
	Low	91.7	86.6	86.41	84.64	82.75	86.42
Visual range	Near-field view	92.69	89.63	88.5	86.18	84.15	88.23
	Far-field view	91.85	86.97	86.19	84.6	82.46	86.41
Space	Indoor	93.27	88.8	87.95	86.33	84.42	88.15
	Outdoor	92.44	86.86	86.17	84.75	82.21	86.49
Overlap of worker	Non-overlap	93.16	88.72	87.62	86.8	84.82	88.22
	Overlap	92.36	86.86	85.65	84.73	82.49	86.42
Density of worker	Scatter	92.78	89.15	87.69	86.86	84.21	88.13
	Grouped	92.32	86.97	86.18	84.7	82.34	86.51
Number of workers	Single	93.45	89.26	88.42	86.68	84.38	88.44
	Mutiple	92.41	86.57	85.68	84.03	82.31	86.2

Additionally, scenarios involving multiple workers or significant overlap considerably reduced accuracy, indicating the model’s sensitivity to visual clutter and partial occlusion. Indoor scenarios generally achieved slightly higher accuracy (mAP: 88.15%) compared to outdoor environments (mAP: 86.49%), likely due to better-controlled visual backgrounds and lighting. These findings underscore practical challenges encountered in real-world construction environments, emphasizing the need for robust solutions tailored to handle varying illumination, object scale, and crowded scenarios.

### Comparative analysis

#### Experiments with benchmark datasets

##### Data description

**Fig. 11:**
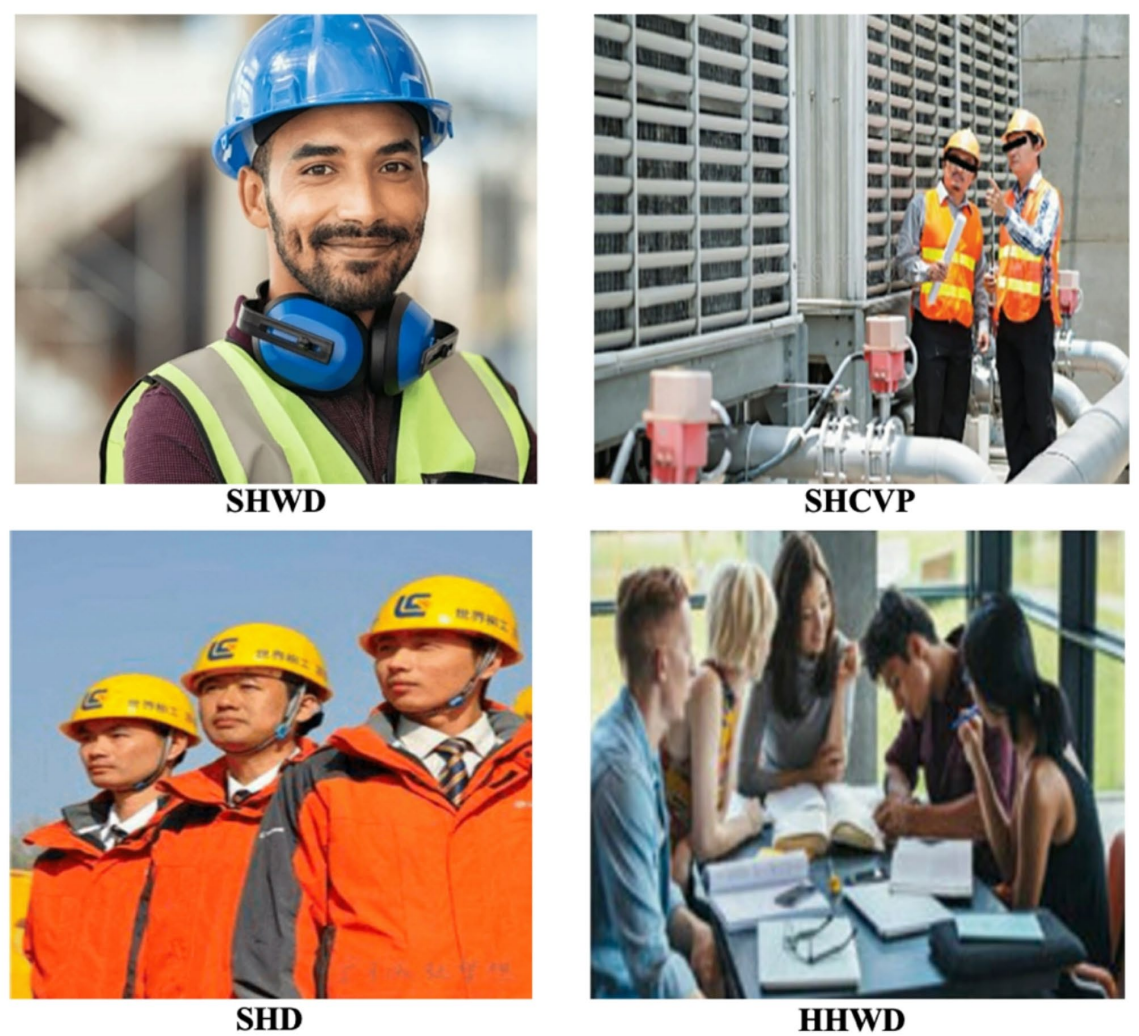
Visual example of each benchmarking dataset.

As presented in Table 10, four widely used benchmark datasets^39–42^ were employed to evaluate the models developed in this research. Most existing benchmark datasets are not exclusively derived from construction sites; instead, they often include images collected from search engines such as Google or Baidu, resulting in scenarios that may not accurately represent real-world construction environments. Additionally, these datasets typically include only non-safety helmet images, accompanied by a limited number of images from other non-PPE classes. Consequently, evaluations conducted using these datasets were restricted to non-helmet tests only. Illustrative examples from these datasets are presented in Fig. 11.

**Table 10:** Description of benchmark dataset.

Dataset name	Number of images	Numbers of non-safety helmet
Safety Helmet Wearing-Dataset (SHWD) ^39^	832	921
Safety Helmet Computer Vision Project (SHCVP) ^40^	1,321	1,625
Safety Helmet Detection (SHD) ^41^	1,537	1,011
Hard Hat Workers Dataset (HHWD) ^42^	1,135	837

##### Comparison results with benchmark datasets

Table 11 presents the performance results of the trained model when evaluated on external benchmark datasets (SHWD, SHCVP, SHD, and HHWD). The highest detection performance was observed on the original dataset (Our dataset), achieving an AP50 of 92.4%, AP50:95 of 68.23%, and notably high IOU of 87.46%. However, when tested on the external datasets, the model exhibited a noticeable reduction in accuracy, with AP50 declining by approximately 4–7% points. Specifically, performance metrics ranged from 85.9% (SHD) to 88.23% (SHCVP) for AP50, and from 60.38% (SHWD) to 62.59% (SHD) for AP50:95.

This performance drop likely reflects domain shifts between the original dataset and external benchmarks, including variations in viewpoints, lighting conditions, object scales, and environmental factors. Smaller objects (AP_S) consistently demonstrated more significant accuracy reductions, highlighting specific challenges related to object scale and perspective variations. These findings emphasize the necessity of incorporating a broader diversity of training examples representing various viewpoints and environmental contexts to enhance the model’s robustness and generalization capabilities in practical, real-world scenarios.

**Table 11:** Performance of trained model on different datasets.

Dataset	Metric					
	AP50	AP50:95	IOU	$\:{\text{A}\text{P}}_{\text{S}}$	$\:{\text{A}\text{P}}_{\text{M}}$	$\:{\text{A}\text{P}}_{\text{L}}$
Our dataset	92.4	68.23	87.46	64.78	89.52	96.71
SHWD	87.28	60.38	82.26	57.39	85.05	94.4
SHCVP	88.23	60.63	82.66	56.95	85.46	92.77
SHD	85.9	62.59	83.91	59.05	84.61	93.66
HHWD	87.1	62.36	82.62	59.22	84.64	93.98

#### Comparison with other detectors

##### Selection of other detectors

To demonstrate the superiority of the proposed YOLOv10 model utilizing a Swin Transformer backbone, comparative evaluations were conducted against several widely-used object detection architectures, including Faster R-CNN, SSD500, and Region-based Fully Convolutional Network (R-FCN). These models employed various alternative backbone architectures such as ResNet-101, ResNet-152, and MobileNetv3. Additionally, YOLOv10 with CNN-based backbones (CSPNet, ConvNeXt, EfficientNet), YOLOv5 (CSPDarknet53s, CSPDarknet53x), and YOLOv8 (Swin Transformer, PVT, Axial Transformer) were included to provide a comprehensive comparison.

All experimental setups, including batch sizes and iteration counts, were kept consistent with their original configurations to ensure fairness in comparisons. Due to unreported hyperparameter optimizations and omitted architectural details in existing studies, exact replication was challenging; therefore, this research implemented methods from previous studies as closely as possible to facilitate a fair comparative analysis.

##### Comparison results with other detectors

Table 12 presents a comprehensive comparative evaluation of the proposed method against other detection models on the test dataset. The proposed YOLOv10 model utilizing a Swin Transformer backbone consistently demonstrated superior performance across key accuracy metrics—AP50 (87.32%), AP50:95 (64.71%), and IOU (82.39%)—compared to other popular detectors and backbone combinations.

Specifically, the proposed method outperformed competing models by approximately 1–3% points in AP50 and about 0.5–1% points in AP50:95. Additionally, it achieved the highest AP across various object scales (small: 61.22%, medium: 84.9%, large: 91.51%), highlighting its robust detection capability irrespective of object size or complexity.

Regarding inference speed, the proposed model attained a competitive FPS (35.32), slightly faster than lighter CNN-based architectures such as SSD500 with ResNet-152 (32.84 FPS) and YOLOv10 with EfficientNet (34.01 FPS), and notably faster than heavier CNN-based architectures like Faster R-CNN with ResNet-101 (20.77 FPS). YOLOv8 with Swin Transformer and YOLOv10 with CSPNet achieved comparable speeds (31.90 FPS and 33.12 FPS, respectively) but exhibited somewhat lower detection accuracy. The detailed FLOPs and parameter counts (in millions) for each method are summarized in Table 7.

Overall, this comparison clearly highlights that the proposed YOLOv10 with a Swin Transformer backbone achieves an optimal balance between detection accuracy and real-time efficiency, making it particularly suitable for high-precision, real-time detection tasks in construction-site environments.

**Table 12:** Comparative results of applying the proposed method and other models on test dataset.

Detector	Architecture	Metric						
		AP50	AP50:95	IOU	$\:{\text{A}\text{P}}_{\text{S}}$	$\:{\text{A}\text{P}}_{\text{M}}$	$\:{\text{A}\text{P}}_{\text{L}}$	FPS
Faster R-CNN	ResNet-101	85.88	61.83	80.06	59.22	84.01	90.62	20.77
	ResNet-152	84.65	62.71	80.12	60.67	81.98	88.93	22.82
	MobileNetv3	86.37	63.75	81.13	59.41	83.32	90.28	28.14
SSD500	ResNet-101	86.47	63.48	80.97	59.58	82.44	90.51	26.84
	ResNet-152	85.34	64.09	80.37	60.29	84.24	88.64	32.84
	MobileNetv3	84.81	63.45	81.65	59.01	83.3	90.7	26.59
R-FCN	ResNet-101	86.73	61.94	81.24	59.06	83.62	89.71	27.27
	ResNet-152	86.36	61.79	79.95	58.37	82.16	89.52	32.26
	MobileNetv3	86.6	63.72	81.78	59.91	83.43	90.33	31.02
YOLOv5	CSPDarknet53s	86.12	63.65	81.27	59.35	82.78	90.13	32.90
	CSPDarknet53x	86.70	63.92	81.54	60.02	83.20	90.45	30.45
YOLOv8	Swin Transformer	86.89	64.10	81.77	60.34	83.68	90.55	31.90
	PVT	86.58	63.87	81.62	59.89	83.45	90.41	32.72
	Axial Transformer	86.43	63.70	81.49	59.64	83.18	90.37	32.10
YOLOv10	CSPNet	86.94	64.21	81.96	60.58	83.82	90.72	33.12
	ConvNeXt	86.77	64.05	81.82	60.74	83.45	90.90	32.48
	EfficientNet	86.55	63.88	81.68	60.42	83.24	90.68	34.01
Proposed method		87.32	64.71	82.39	61.22	84. 9	91.51	35.32

## Conclusions

This research conducted an extensive evaluation of YOLOv10-based object detection models tailored specifically for automated detection of non-PPE compliance on construction sites. The focus was placed on identifying critical PPE items, including helmets, masks, vests, gloves, and shoes. The analysis utilized a diverse dataset compiled from construction site surveillance cameras, body-worn camera recordings, and publicly available benchmark sources to rigorously assess PPE compliance scenarios.

Due to initially limited data availability for the non-safety helmet category, image augmentation techniques were selectively applied only to images containing non-safety helmets to enhance dataset balance. In evaluating the effectiveness of these augmentation methods, the ViT-based model demonstrated significant performance gains; however, the best overall results were achieved by the Swin Transformer-based YOLOv10 model. Specifically, the Swin Transformer model recorded AP50 metrics of 92.4% for non-helmet, 88.17% for non-mask, 87.17% for non-vest, 85.36% for non-glove, and 83.48% for non-shoes, with an average AP50 of 87.32%.

Experimental findings also confirmed the superior performance of transformer-based architectures compared to traditional detection frameworks such as Faster R-CNN, SSD, and R-FCN utilizing various backbone architectures. The consistent accuracy, notably high for essential PPE items like helmets, indicates that the Swin Transformer model is highly suitable for real-world safety monitoring, where reliability and accuracy are paramount.

Despite these promising outcomes, certain limitations were identified. One key limitation involves reduced detection accuracy for smaller PPE, as highlighted by relatively lower AP_S scores across datasets. This indicates a particular challenge in identifying helmets appearing small due to distance or lower image resolution—an issue critically relevant in crowded or expansive construction environments. Additionally, the performance decrease observed when models were tested on benchmark datasets indicates challenges related to generalizing effectively across diverse data distributions and unfamiliar conditions.

For future studies, enhancing model generalizability remains crucial. This can be accomplished by expanding training datasets to include greater image diversity, covering various environmental conditions, camera viewpoints, and scenarios beyond typical construction settings. Moreover, considering the challenges identified by the authors regarding small object detection, previous research in different contexts^43,44^ has addressed similar issues by incorporating additional modules, such as super-resolution techniques, into YOLO. Therefore, further exploration into integrating these hybrid approaches with transformer architectures is warranted.

## Data Availability

The datasets generated during and/or analysed during the current study are available from the corresponding author on reasonable request.
